# Are Brazil’s Deforesters Avoiding Detection?

**DOI:** 10.1111/conl.12310

**Published:** 2016-11-07

**Authors:** Peter Richards, Eugenio Arima, Leah VanWey, Avery Cohn, Nishan Bhattarai

**Affiliations:** 1 Institute at Brown for Environment and Society, Brown University, Providence, Rhode Island, USA; 2 Bureau for Food Security, United States Agency for International Development, Washington, D.C., USA; 3 Department of Geography, University of Texas, Austin, Texas, USA; 4 The Fletcher School, Tufts University, Medford, Massachusetts, USA

**Keywords:** Brazil, Amazon, deforestation, greenhouse gas emissions

## Abstract

Rates of deforestation reported by Brazil’s official deforestation monitoring system have declined dramatically in the Brazilian Amazon. Much of Brazil’s success in its fight against deforestation has been credited to a series of policy changes put into place between 2004 and 2008. In this research, we posit that one of these policies, the decision to use the country’s official system for monitoring forest loss in the Amazon as a policing tool, has incentivized landowners to deforest in ways and places that evade Brazil’s official monitoring and enforcement system. As a consequence, we a) show or b) provide several pieces of suggestive evidence that recent successes in protecting monitored forests in the Brazilian Amazon may be doing less to protect the region’s forests than previously assumed.

## Introduction

According to the Program for the Estimation of Deforestation in the Brazilian Amazon (PRODES) deforestation monitoring system, forest loss in Brazilian Amazon dropped from more than 25,000 km² in 2003/04 to an average of 5,200 km² year between 2009 and 2013 (INPE 2015). This decline has been widely hailed as a success story in environmental policy in Brazil (Walker *et al.* 2009; Nepstad *et al.* 2014; Soares-Filho *et al.* 2014; Gibbs *et al.* 2015). Unofficial indicators of forest loss, however, do not corroborate this trend.

In this research, we show that in 2008, after the Brazilian government began using PRODES for as part of enforcement activities, observations of deforestation dropped significantly in PRODES but not in two two other systems traditionally closely correlated with deforestation . We posit that this divergence owes to a transformation in the use of PRODES, which may have led to a change in landowners’ clearing strategies. We estimate that as much as 9,000 km² of forest loss since 2008 may have leaked to areas unobserved by PRODES, due to changes in how landowners viewed and responded to the PRODES system.

## Governing forest loss

Starting in 2004, government and nongovernmental groups began implementing numerous interventions to reduce deforestation in the Brazilian Amazon. Over the 2004–2007 period, the Federal Government of Brazil implemented the first phase (i) of the Plan for Preventing and Controlling Deforestation in the Amazon (PPCDAm). PPCDAm i created vast expanses of protected areas (Soares-Filho *et al.* 2006; Arima *et al.* 2014); a new agency to manage them (Instituto Chico Mendes); a restructured Brazilian Environment and Natural Resources Institute (IBAMA), to focus exclusively on enforcement and regulation; and initiated the use of “real-time” deforestation data to investigate new clearings (Brazil 2013). Around the same time, in response to the rise in deforestation rates earlier in the decade, private companies and Non Government Organizations (NGOs) came together to organize a series of interventions to limit deforestation associated with the soy, beef, and timber supply chains. These interventions, combined with a shift in market favorability for key land uses in the Amazon, led to a substantial drop in deforestation after 2006 (Nepstad *et al.* 2014; Gibbs *et al.* 2015).

In 2008, in response to a slight rise in deforestation rates, the Brazilian government initiated a second phase of PPCDAm. PPCDAm ii offered new tools for environmental monitoring and enforcement (Assunção *et al.* 2012; Abman 2014). This included using the widely regarded PRODES system as a tool for enforcing environmental laws in the Amazon. Under PPCDAm ii, counties with high levels of forest loss (as measured through PRODES) were subject to credit restrictions (Duchelle *et al.* 2014). PRODES deforestation data were also matched with property data to identify the owners of newly cleared areas, or individuals who violat local environmental laws. Thereafter, the owners of land observed as deforested in PRODES could face fines, property embargoes, or even imprisonment (Assunção *et al.* 2012; Arima *et al.* 2014).

PPCDAm ii had a substantial and near immediate effect on deforestation as measured by the PRODES system—deforestation fell from 12,000 km² in 2008 to just 5,000 km² per year in each year since. The program has been hailed for reducing deforestation in the Brazilian Amazon (Assunção *et al.* 2012; Arima *et al.* 2014). One source of suggestive evidence of the effect of PPC-DAm ii on deforestation has been the rise of deforestation in other regions of Brazil (Morton *et al.* 2016) and neighboring South American nations over the same period (Graesser *et al.* 2015; de Waroux *et al.* 2016). However, the incomplete scope and extent of forests and deforestation monitored by PRODES means that there was also a potential for an additional response to enforcement, namely, deforestation tailored to avoid detection by PRODES. PRODES has never monitored dry or secondary forests in the Amazon Biome that are nor does it monitor cleared patches less than 6.25 ha (Souza Jr *et al.* 2003; Asner *et al.* 2005; Hansen *et al.* 2008; Broich *et al.* 2009; Rosa *et al.* 2012; INPE 2013; Souza Jr *et al.* 2013; Toomey *et al.* 2013). In the following section, we show after 2008 deforestation shifted those areas which are not actively monitored by Brazil’s PRODES system.

## Multiple comparisons show divergence between PRODES and other deforestation indicators after 2008

If transformating PRODES into a tool for identifying deforesters and enforcing environmental law affected its ability to provide a consistent measurement of forest loss, then (1) PRODES deforestation rates should correlate with other metrics before 2008, but diverge after 2008; and (2) divergence should be greatest in areas where landowners would be most aware of the PRODES system and its importance for enforcing environmental laws, and have the greatest incentive to avoid detection. We show evidence of both of these trends through several analyses. In this section, we compare annual trends in deforestation data in PRODES versus two other datasets (the Global Forest Change [GFC] dataset [Hansen *et al.* 2013] and the Fire Information for Resource Management System [FIRMS 2015]); analyzing where, when, and how these datasets diverge; and examine the spatial patterns that underlie their divergence.

## PRODES deforestation diverged from other deforestation indicators after 2008

From 2002 to 2008, PRODES estimated that, on average, approximately 19,000 km² of forests were lost annually in the Amazon Biome. Deforestation was highest from 2002 to 2005, when forest loss rates exceeded 20,000 km² per year. Rates then fell over the course of 2006–2008 to approximately 10,000 km². After PPCDAm ii, they fell even further to 5,000 km² per year.

From 2002 to 2008, the GFC data estimated that, on average, about 20,000 km² of forests were lost per year. Just as with PRODES, GFC recorded the highest forest loss rates during the early part of the decade. Rates then dropped in 2006 and 2007, to approximately 15,000 km² per year. However, loss rates did not drop to the same extent as the PRODES estimates after 2008. From 2009 to 2013, deforestation rates in the GFC data remained around 10,000 km² per year, or roughly double PRODES levels. Significant deforestation spikes occurred in 2010 and 2012, when loss rates increased to approximately 15,000 km² per year.

The FIRMS data follow the GFC data. Fires were prolific during the early 2000s. The number of fire incidents reached a nadir in 2005, but fell to lower levels in 2006. From 2009 to 2013, the number of fires recorded per year fell, on average, fell to less than half of levels observed earlier in the decade. Significant spikes in fire incidents were observed in 2010 and 2012, the same years for which deforestation spikes were observed in the GFC data.

A paired *t*-test of annual differences in deforestation in the Amazon Biome revealed no significant difference between the PRODES and GFC data for the years 2002–2008 (*t* = 0.73; df = 6, *P* = 0.49). The same test revealed a sharp divergence in the datasets over the period 2009–2014 (*t* = 5.19; df = 4; *P* = 0.0066). The GFC and FIRMS data show similar trends over this latter period.

## Sources of PRODES divergence

**Figure 1:**
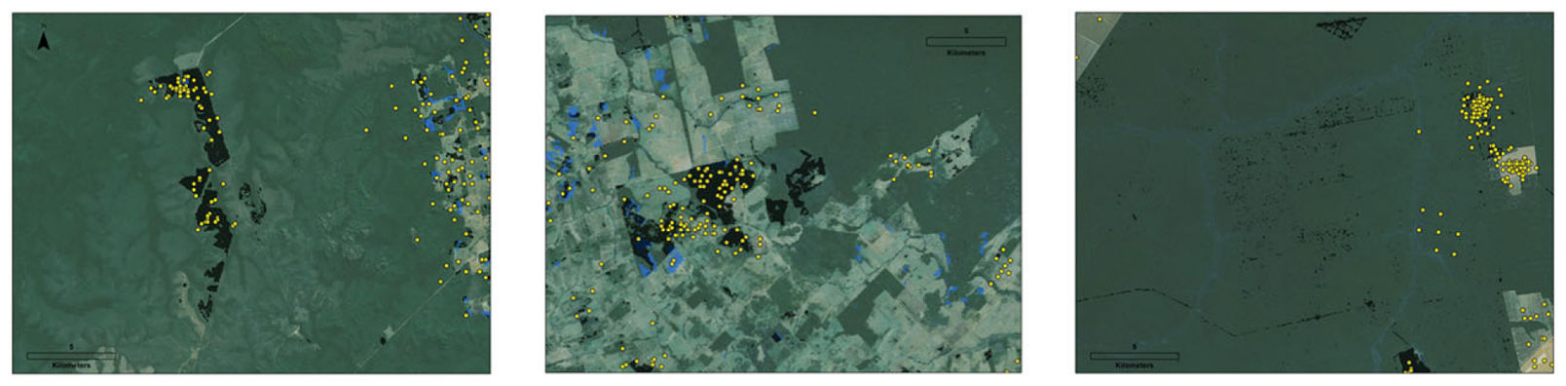
Deforestation and fire in Mato Grosso, Brazil Close up examples of land clearing classifications in three areas of Mato Grosso Brazil. Post deforestation classifications by GFC are shown in black; PRODES deforestation classifications are shown in blue; and FIRMS fire incidents are marked with yellow dots. At left, a large area in the center of the image is burned and marked as deforested in the GFC data, but PRODES records no clearing. In PRODES, this area was marked from monitoring as “nonforest.” At center, an area marked as already deforested in PRODES is classified as burned and cleared in the FIRMS and GFC datasets. At right, small clearings associated with logging are observed in the GFC dataset but not in PRODES. Fire is sparsely used in logging operations, and no fires are observed in these areas.

Approximately 73% of forest loss reported by PRODES was identified as forest loss by the GFC dataset. However, only 51% of total GFC forest loss (2001–2013) was reported in PRODES during the period 2002–2013 (see Supplementary Information). Much of the difference between the GFC and the PRODES classifications stemmed from clearings in small patches, the clearing or destruction of scrub or riverine forests, and secondary forest clearings (Table S10 in Supplementary Information). Annually, per the GFC data, approximately 5,000–6,000 km² of forest was deforested in plots smaller than 6.25 ha, the minimum threshold for inclusion in the PRODES-based statistics (Table S4). Deforestation rates in the drier portions of the biome, or in riverine areas, also remained steady across the entirety of the time period. In total, we estimated that about 500 km² of GFC forest loss occurs each year in areas which PRODES preclassifies nonhumid forest. Another 2,500 km² of forest is cleared in areas classified as already deforested in the PRODES data. Deforestation in these areas is not deterred by PRODES, given that these areas are not monitored under the PRODES system (see Figure 1 for several illustrations of these clearings).

## Locations of PRODES divergence

Several clear spatial differences emerged when comparing the GFC and PRODES data. To highlight these differences, we aggregated deforestation pixels from both the PRODES and GFC data into a layer of 30 km × 30 km grid cells (*n* = 4,931). Had the divergences between the GFC and PRODES datasets been attributable to systemic error, the differences would be randomly distributed across the region. The differences between the PRODES and GFC data, however, are clearly concentrated in several key regions.

**Figure 2:**
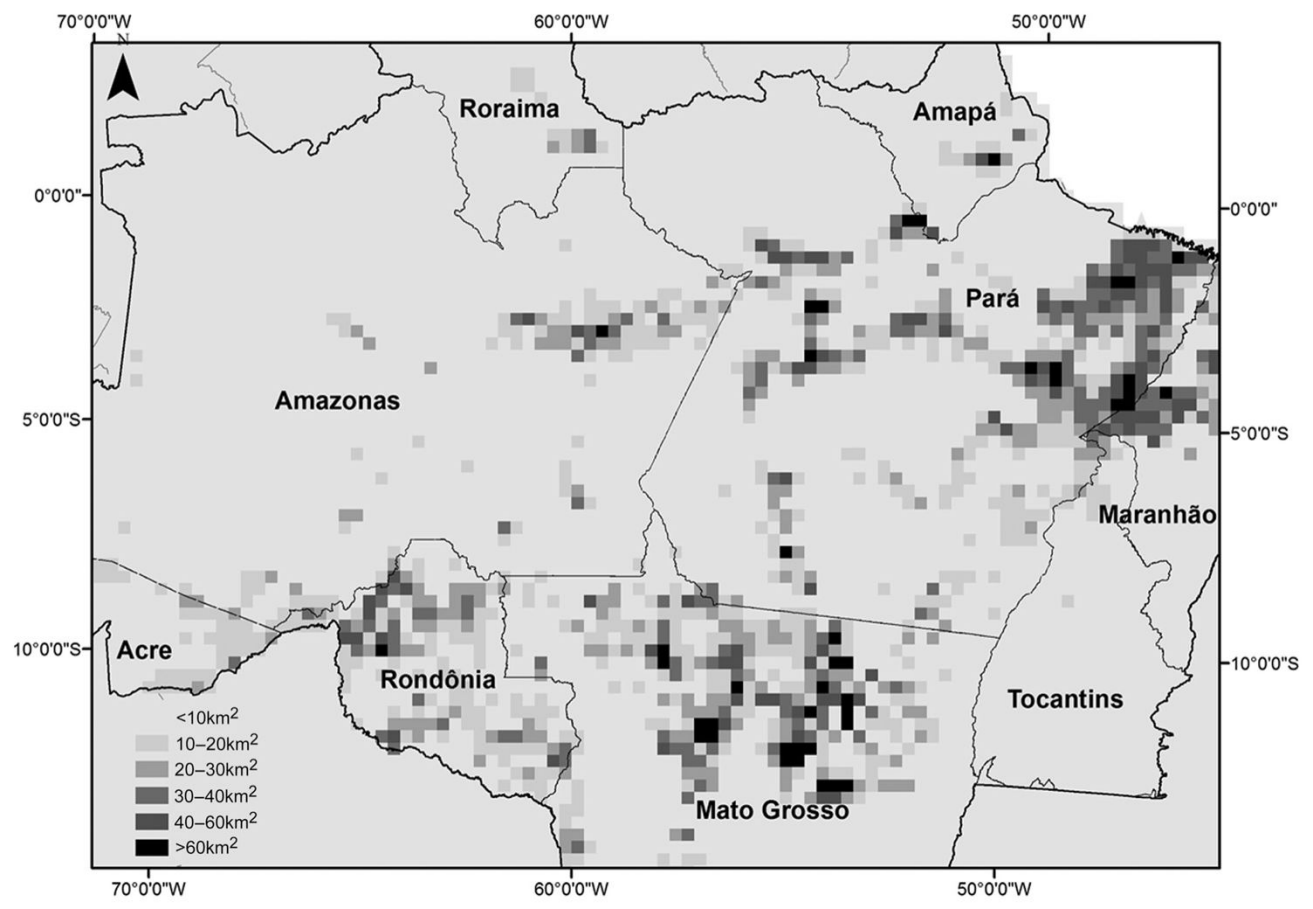
Spatial distribution of differences between deforestation observed in GFC and PRODES deforestation classifications after 2008 Differences in total observed deforestation, in 30km x 30km grid cells, in GFC and PRODES data from 2009–2013. Darker shades indicate the largest levels of discrepancies between the official and unofficial deforestation indicators. The greatest differences are found in northeast Pará State and north-central Mato Grosso.

The largest discrepancies between the GFC and PRODES data were found in northern Mato Grosso, where a thriving soybean sector is creating high demand for land; and in northeastern Pará, where investments in cattle processing, soybean production (in the region around Paragominas), and palm oil production are transforming the region into one of the most rapidly growing rural economies in the Amazon (Figure 2). We argue that these areas are those where landowners would have both the greatest incentives to avoid detection, and be more likely to have knowledge on how to avoid the monitoring system.

In the mid-2000s, the States of Pará and Mato Grosso began requiring large landowners to register their properties in their respective Rural Environmental Registries, known more commonly as CAR (Chomitz & Wertz-Kanounnikoff 2005; Azevedo & Saito 2013; Richards & VanWey 2016). To register in the CAR system, property owners needed to create geospatial information on property boundaries and forest cover. To accomplish this task, many landowners sought the help of technical experts knowledgeable in geospatial data. In addition to creating the data needed for the CAR, these experts also needed to understand local environmental laws and the official forest classifications. By extension, we would expect that many of the more-capitalized farms in Mato Grosso and northeastern Para, two area which would have had been more likely to register in the CAR system, would have had more knowledge of the country’s monitoring systems, and more importantly, a better understanding of how their land (and potential land acquisitions) was classified in PRODES.

These same landowners would have also had a stronger incentive to open new lands. Opening new land has long been seen as a key means for increasing property values. The greatest returns to opening new land may be in these higher valued regions in north-central Mato Grosso and in northeastern Pará. Landowners in these areas, presumably, would thus have had both the greatest incentive to continue opening land and more awareness with respect to which lands could be opened without triggering a deforestation observation. Smallholder farmers and ranchers, in contrast to their more capitalized counterparts, may not have had access to the same technical knowledge. They also would have had less incentive to avoid deforestation detection. Small farming areas are less likely to be subject to environmental enforcement, despite higher rates of forest loss (Godar *et al.* 2014; Schneider & Peres 2015; Richards & VanWey 2016).

## Implications for deforestation accounting

**Figure 3:**
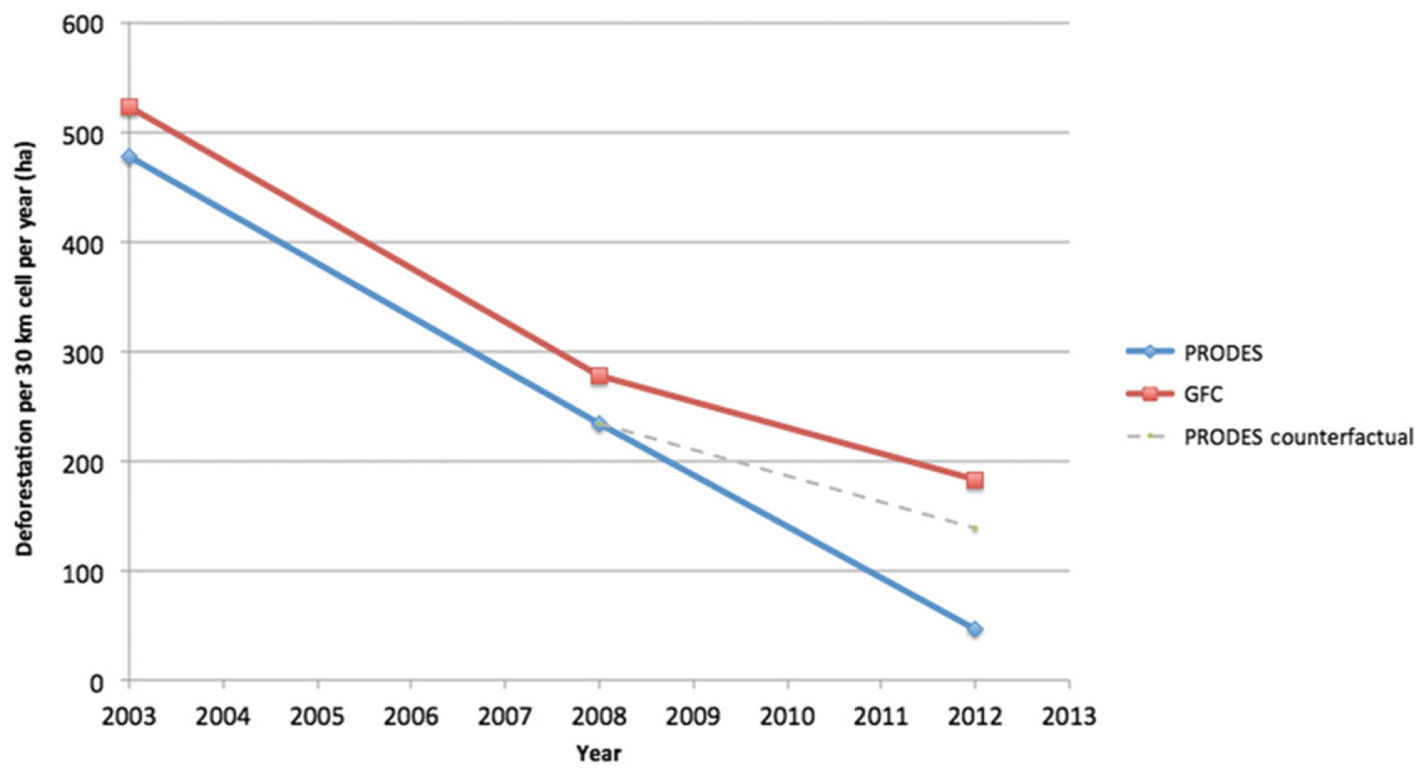
PPCDAm ii caused PRODES to underestimate 900,000 ha of deforestation A differences-in-differences analysis revealed PRODES and GFC to have had similar trends in deforestation between 2003 and 2008, but that PRODES deforestation declined while GFC deforestation did not post 2008. The finding is consistent with our hypothesis that deforesters sought to avoid PRODES, but not GFC monitoring. The red segments depict the GFC deforestation rate over the two periods as predicted by our statistical analysis. The blue segments depict the PRODES deforestation rate over the two periods as predicted by our statistical analysis. The gray segment depicts a counterfactual of the PRODES deforestation rate over the period 2008–2012 had it been the same as the GFC rate over the period. The results of the counterfactual simulation reveal an estimated discrepancy of greater than 900,000 ha over the period.

To estimate the amount of deforestation missed by PRODES, we performed a differences-in-differences analysis of deforestation levels using the layer of 30 km × 30 km grid cells. The analysis reveals a significant negative bias effect on PRODES deforestation in years following 2008 (*P*<0.01). This analysis also, offer a counterfactual measure of deforestation, or a proxy estimate deforestation “lost” due to the new enforcement application of PRODES. In total, we estimate that nearly 9,000 km² of deforestation was missed by the PRODES system, or due to local-level incentives to avoid observation. This area corresponds to an area roughly the size of Puerto Rico (See Figure 3; full regression results and alternative specifications are included in the Supplementary Information).

## PRODES-derived greenhouse gas emissions estimates increasingly inaccurate

PRODES is used as the basis for greenhouse gas emissions estimates for Brazilian forest loss, and thus an important part of Brazil’s climate change mitigation policy. A downward distortion in deforestation levels therefore carries implications for Brazil’s greenhouse gas emissions. To illustrate the implication of using PRODES, as opposed to an unofficial and universal indicator of forest loss as the basis for emissions accounting, we estimated post-2008 emissions using both the GFC and PRODES datasets, and two widely cited measures of above ground live biomass: the Amazon Basin Aboveground Live Biomass distribution map (Saatchi *et al.* 2009) and the Pantropical National Level Carbon Stock dataset (Baccini *et al.* 2012).

Total emissions from Amazon deforestation based on the GFC data are nearly twice as high as estimates based on PRODES. GFC-based greenhouse gas estimates suggested that more than 500Tg of carbon was released through deforestation in the Amazon Biome over the 2009–2013 period. Estimates using the same methods and the PRODES data suggested approximately 250Tg of emissions (see Supplementary Information). Estimated losses in emissions *per hectare*, however, were significantly higher for PRODES statistics (93.2–97.9 t/ha) than for the GFC data (82.5–84.8 t/ha). This should be expected given that the PRODES data only consider deforestation of relatively biomass-rich regions, while the GFC data account for forest loss on relatively biomass-poor secondary and scrub forests.

## Conclusion

PRODES does an admirable job of meeting its objectives, albeit with some well-established technical shortcomings (Asner *et al.* 2005; Souza Jr *et al.* 2013). Using PRODES as a foundation for regulating forest loss in the Amazon has also likely helped to deter the clearing of large patches of primary forests. However, in this research, we show that some of this deforestation has simply shifted to other portions of the Amazon Biome not monitored by PRODES. We thus suggest that, since 2008, PRODES monitored deforestation has become less representative of all deforestation in the Brazilian Amazon and therefore has become less accurate as a component of the system Brazil uses to estimate Greenhouse Gases (GHG) mitigation from avoided deforestation.

This article is not a specific critique of PRODES nor an argument that GFC (Tropek *et al.* 2014) or the FIRMS would be suitable replacements; rather, it is a general warning about the increased challenges to accurate monitoring of deforestation in the presence of strong enforcement activities reliant on the same monitoring scheme. We expect that the more stringent the enforcement tied to PRODES, the less accurate the PRODES estimate of annual forest loss can be expected to be. Thus, as enforcement tightens to meet Brazil’s GHG and deforestation policy objectives, we expect that PRODES forest loss estimates will become progressively less accurate as the basis for Brazil’s accounting of GHG emissions from deforestation.

We see two solutions to the problem we have identified. First, the development of next-generation deforestation monitoring systems with more reach, higher resolution, and better accuracy will be critical, especially for emissions monitoring. Notably, many of the limitations on data processing, spatial resolution, and data access which the original architects of PRODES once faced no longer exist. Landsat 8 features newer and higher resolution imagery, and ultrahigh-resolution data are becoming increasingly available at greater temporal frequencies. Forest loss monitoring in the Amazon could be updated to be made more dynamic with respect to reporting (1) nonanthropogenic forest loss, (2) the loss of secondary forests, (3) the forest loss in the drier portions of the basin, and (4) forest degradation, or forest loss in small clearings. Such rich detail on land use, land use transitions, and greenhouse gas emissions appears in Brazil’s recently-published third national inventory of greenhouse gas emissions and sequestrations (Brazil 2016). However, these inventories do not provide the high temporal frequency and real-time data that are the hallmarks of the PRODES system. Second, monitoring systems and enforcement mechanisms should be separated. Tying the enforcement of environmental laws explicitly to a tool for scientific monitoring distorts landowners’ clearing incentives, and pushes clearing activities to areas less likely to be observed. This has the perverse impact of overstating any deforestation reductions associated with new enforcement tools.

Transparently achieving Brazil’s GHG mitigation commitments will require more than the antiquated and incomplete approach to tropical forest monitoring that is the status quo. Our findings suggest that a focus on large patches of primary forests is likely to leave Brazil far from truly ending deforestation and far from ready to transfer a system for ending tropical forest loss to other developing countries. While Brazil can rightly celebrate the reduction in large-scale clearing of primary forest, continued clearing threatens the country’s ambitious targets. It is time to develop a new system that tackles the problem of illegal deforestation comprehensively by monitoring the cerrado, secondary forests, and the persistent number of small clearings.

## Supplementary Material

Supplemental materials
