# Supplementary material for: Are Brazil’s Deforesters Avoiding Detection?
Source: Conserv Lett. Author manuscript; Available in PMC 2017 Dec 19. (PMC5736146; doi:10.1111/conl.12310)
Supplement: Supplemental materials [file NIHMS867509-supplement-Supplemental_materials.pdf]

SUPPLEMENTAL MATERIALS

Brazilian Policies on Deforestation

Brazilian policies to control deforestation in the Amazon comprise multi-level strategies ranging from the landscape to the private property scale. Prior to 1995, environmental policy in Brazil relied mostly on the creation of protected areas (*1*) and on the 1965 Forestry Code, which stipulated that land owners in Amazonia should keep 50% of their private properties as a forest reserve, known as legal reserve. The code also required landowners to preserve areas along rivers, steep slopes, and top of hills. In 1996, the legal reserve requirement was increased to 80%, a number that remains with the new Forestry Code, approved in 2012. Another important legislation is the Environmental Crimes Law, enacted in 1998, that defines the crimes against Brazil's fauna and flora and stipulates punishments for violations.

Enforcement of these environmental laws had little traction until 2004 when the Brazilian government enacted the first Action Plan to Prevent and Control Deforestation in Legal Amazonia (PPCDAm – I). Implemented between 2004-2007, this plan created a new agency to foster and manage Brazil's protected areas (Instituto Chico Mendes) and restructured the environmental agency's mission (IBAMA) to focus exclusively on enforcement and regulation. IBAMA also began using INPE's 'real-time' deforestation detection (DETER) to target is enforcement efforts in the field (*2*). Another component of the plan was the expansion of the protected area system. Since 2004, 25 million hectares of federal conservation units, 10 million hectares of indigenous lands, and 25 million hectares of state conservation units were added to the system of protected areas in Amazonia (*3, 4*).

The second phase of PPCDAm (2008-2011) focused on monitoring and enforcement of the environmental legislation. PPCDAm-II originally placed 36 municipalities on a list for special enforcement efforts due to historically high deforestation rates. This list was later expanded to 43 (2009) and 48 municipalities (2011) after two were removed. In 2012, two more municipalities were added and four removed, leaving 46 as of today. Municipalities on the list were subjected to higher levels of law enforcement and increased number of environmental fines. Removal from the list is contingent upon sustained reduction of deforestation rates, creation of georeferenced cadastral maps of private properties, and plans for restoring areas deforested illegally in each property (*2, 4*). Complementing those efforts, Federal Prosecutors (Ministério Público Federal) initiated in 2009 civil actions against meat packing plants purchasing cattle from non-compliant farms and offered to suspend those actions if companies agreed to purchase cattle only from ranchers that followed the directives established by PPCDAm(*5*).

PPCDAm-III (2012-2015) continues its focus on monitoring, control, and enforcement of environmental laws and adds policies to improve the governance over the territory through land titling, combat against illegal ownership of public unclaimed lands ('grilagem'), and implementation of ecological-economic zoning. PPCDAm-iii also aims at supporting forest valuation mechanisms (e.g. REDD+, payment for ecosystem services), better use of areas already deforested, and supply-chain processes (*2*)

DATA

We use four key data sources for our analysis: [1] Projeto de Monitoramento do Desmatamento na Amazônia Legal por Satélite (Monitoring Deforestation in the Legal Amazon by Satellite, or PRODES), produced annually by National Institute for Space Research (*6*), [2] the Global Forest Change (GFC) dataset *(7),* [3] the Fire Information for Resource Management System (FIRMS) data (*8*), and [4]

the MCD45A1 collection 5.1 burned area product (*9*) . Prior to analyzing the data we projected each dataset into an Albers equal area projection. We also reclassified the PRODES data into 30m resolution pixels, the equivalent of the GFC product. We focus on the period 2002-2013.

PRODES

PRODES is based on landsat 5/TM images and is used as the basis for Brazil's official estimates of forest loss. PRODES was initiated in 1988, and has been in continuous use for more than twenty-five years. PRODES maps *corte raso* deforestation, or clear cut forest loss occurring over a brief period of time, in areas larger than 6.25ha. It does not record degradation or forest loss attributable to logging. PRODES has been freely available for download since 2002 (*10*).

Deforestation rates in PRODES are calculated based on land use change between dry seasons. The imagery used in the classifications is acquired between June and August of each year (the dry season). Areas cleared in the early rainy season would thus be classified as deforested during the following classification cycle. To be classified as deforested, the area cleared must be larger than 6.25ha and classified as deforestation due to *corte raso*, or due to an anthropogenic cutting and clearing of the forest. As stated in the main text, however, the PRODES classifications are applied only to areas that are not pre-classified as (a) water, (b) non-forest, (c) or previously deforested. Each year's classifications are thus based in part on the prior year's PRODES product.

Since 2008, PRODES classifications have come to occupy a key role in policy decisions regarding the Amazon. Plans to reduce forest loss by 80% over 2005 levels, as determined in the 2009 United Nations Climate Change conference were predicated on targets and baselines set and achieved with respect to the PRODES indicators. The Norwegian donation to Brazil's Amazon Fund was contingent on Brazil's progress toward meeting these goals. Since 2008, access to federal credit and property regularization in the Amazon have likewise been conditional on deforestation rates recorded by PRODES (*10*).

We only analyze land use change within Brazil's Amazon Biome; estimates of PRODES-classified deforestation therefore differ slightly the official statistics, which include portions of the Amazon Basing lying outside of the Amazon Biome.

GFC

The Global Forest Change data product is distributed through the University of Maryland and freely viewable through Google's Earth Engine platform. The Global Forest Change Dataset is based on 30m resolution Landsat data, and classifies any areas with vegetation taller than 5m as forest. Deforestation was defined as a "stand-replacement disturbance" and classified by year. Degradation, insofar as it did not lead to a "non-forest state," is not classified as deforestation. The GFC also classifies forest regrowth, although we do not use this indicator in our analysis (*7*). The GFC classification system uses Google's Earth Engine cloud platform, where it integrates the platform's large scale computational capacity with freely available landsat imagery.

GFC data are based on growing season images and were pre-processed based on a prototype method developed for the Democratic Republic of Congo. Classifications ae validated using google earth and other available imagery (*7*).

The GFC dataset has been the subject of some criticism (*11*). This criticism has largely stemmed from defining forest areas based on vegetation heights. Based on this definition, tree plantations (for

example, oil palm) would be classified as forest, as would many secondary forests. Naturally, these areas will not harbor the same level of biodiversity as a mature primary forest. Nor would these areas contain the same level of above ground live biomass. However, given that our analysis focuses on land clearing and its impact on carbon emissions rather than biodiversity, and that we weighted each land clearing by the specific above ground live biomass these issues should not affect our analysis. Finally, we note that Tropek, et al., in their commentary on the Global Forest Change dataset, suggest that the GFC misidentifies a potential soybean farm as forest cover (*11*). This would be of direct relevance to our analysis, given the importance of soybean production to areas of the southern Amazon Basin. However, closer inspection of this area revealed that Tropek, et al. mistook what was a large oil palm plantation as evidence of a misclassification in the GFC data of soybeans for forest.

FIRMS

The Fire Information for Resource Management System (FIRMS) is based on 1km Modis pixels and is produced by the University of Maryland. Each fire location in the FIRMS dataset represents the center of a pixel identified as containing one or more fire. We used the MCD14ML dataset, which identifies both the location and year of each fire incident, from 2001 to present (*8*). The global fire maps are based on Terra and Aqua MODIS sensors from 2001. This dataset was downloaded from the Fire Information for Resource Management System (FIRMS) website.

We note that while fire is a very common method of land clearing, fires also occur naturally, and fire incidents recorded in the FIRMS data may not necessarily lead to forest clearing. Similarly, a set fire may escape a planned burn area and enter areas that will return to forest. Such fires would conceivably be included in our number of fire incidents, even though they do not necessarily lead to deforestation. The drought conditions in 2007 and 2010, for example, likely contributed to the higher number of fire incidents during those years. Nevertheless, fire remains a principal and commonly used method for clearing forests. We thus view the number of fire incidents as a comparative measure for forest clearing activity in the region. In total, the number of fires observed in the Amazon Basin range from more than 300,000 in 2005 to less than 60,000 in 2013.

Burned Area

We also used the MCD45A1 collection 5.1 burned area product, windows 5 and 6. At 500 m resolution, this dataset classifies the degree of confidence in the detection of burn. Pixel values range from 1-5, where 1 denotes highest confidence and 5 denotes detections over agricultural areas as identified by Modis product MCD12 (*9*). Only pixels with value 1 are used in this analysis since those are most likely to be forest fires related to deforestation or degradation. To retrieve those values, we first created yearly burned area layers by taking, for each pixel, the smallest value pixel during the Jan-Dec period of the respective year and then separated the 1's using simple raster algebra. We use the burned area data product exclusively in our meso-scale grid analysis.

Comparability of PRODES and GFC.

The PRODES and GFC datasets were created with different goals and purpose, and thus use slightly different methods in their land use classification. Notably, PRODES was designed specifically for measuring *corte raso* forest loss in the Brazilian Amazon. The present version of PRODES is also based on classification methods developed in the early 2000s. The GFC data was designed to provide

systematic, global coverage at a fine scale resolution. We do not wish to suggest that one dataset constitutes a better product. Rather, each possesses several key advantages.

The key advantage of the GFC data lies in its independence from the Brazilian government (e.g., there is no direct interest in managing the rates of deforestation, and the data is not linked to enforcement), the higher pixel resolution, the continuous coverage, and the more recent baseline for analyzing clearings. However, the classification is fully automated, and is likely to include greater levels of error than the PRODES data. The PRODES data was created exclusively to measure deforestation in primary, humid forests in the Amazon Biome, areas which contain the highest levels of both carbon reserves and biodiversity in the region. However, this design omits the observation of clearings associated with secondary forests or the lower forests in the region.

We also recognize that the PRODES and GFC data are based on images captured during different points in time within a year. The PRODES data is based on the three month window of the Amazon dry season, from June to August, of each year, while the GFC classifications are based on growing season imagery. Given the differences in the classification period, deforestation identified in PRODES may be associated with a previous or later year in the GFC data.

IV Methods and Analysis

We analyzed forest loss trends at three scales. First, we compared the principal datasets based at the biome level. Second, we cross-tabulated the data to analyze pixel-level classification patterns, including patch size. Third, we constructed a meso-scale layer based on an overlay of more than 4,000 900km² grid cells. Finally, and lastly, we then estimate carbon emission based on pixel scale classifications of deforestation in PRODES and GFC.

Biome Level Comparisons

<u>Annual Trends in Forest Loss and Fire Incidents</u>

We estimate deforestation across both the PRODES and GFC data by multiplying the total number of pixels in each classification category by 30*30*0.0001 to convert to hectares. For fire estimates, we count all fire incidents registered in a given year. We include the full results in Table S1. We note that we focus only on the Amazon Biome, rather than the Legal Amazon.

Deforestation in the Amazon Biome, according to PRODES, ranged from 28,000km², in 2004, to slightly more than 4,000km² in 2012. The GFC dataset recorded a deforestation high of 25,500km² of forest loss in 2004, and a low of 9,500km² in 2013. The FIRMS data indicated the highest levels of fire incidents in 2005, when more than 300,000 fire incidents were observed; the lowest number of fire incidents was observed in 2013, at less than 70,000.

Deforestation rates, as observed in PRODES, fell after 2008, or after PPCDAm ii was enacted, and immediately after the first blacklists were imposed in the Amazon region. In the GFC dataset, however, deforestation continues at approximately 10,000km² per year from 2009 through 2013.

<u>Statistical Comparisons of PRODES, GFC, and FIRMS</u>

A key contention of this work is that GFC and FIRMS data correlate with the PRODES data before PPCDAm ii was enacted, but not afterwards. Such a finding would support the argument that landowners were motivated to exploit the weaknesses in the PRODES system after 2008, and then shifted

their land use strategies toward clearing areas that would not be identified as newly deforested in PRODES.

In Figure S1 we graph the distribution of deforestation and fire incidents across the three datasets, both pre and post 2008. The sharp decline in the PRODES data is brought into full relief when contrasted against the relative levels of decline observed in the GFC and FIRMS data. In Table S2 we further explore this relationship by providing a matrix indicting correlations between each of the three key datasets used in this analysis. During the pre-PPCDAm ii period, PRODES and GFC were highly correlated. However, in the post PPCDAm ii period the correlation between both the GFC and FIRMS data and the PRODES indicators falls. Yet at the same time, the correlation between GFC and FIRMS increased, to $r = 0.87$.

In addition to testing correlations across the three key datasets pre and post 2008, we conducted paired t tests on the GFC and PRODES data before and after PPCDAm ii. As indicated in the principal text, we find no significant difference between GFC and PRODES prior to PPCDAm, but significant differences thereafter (see Table S3).

Pixel Level Cross Comparisons of PRODES, GFC, and FIRMS

Cross-Tabulations

We cross tabulated the three principal datasets used in this analysis to identify the sources of variation across the PRODES and GFC estimates. To produce the GFC-PRODES comparisons, we cross tabulated the PRODES land use classification associated with each GFC deforestation location using ArcGIS's zonal statistics tool. We used a slightly different matching technique to cross tabulate the FIRMS data. Rather than using the zonal statistics tool we extracted multi-values to point, then exported the results and tabulated for each year. The results offer insight into the distribution of GFC deforestation and FIRMS fire incidents in the PRODES classification system. We present the results in six tables (Tables S4-S9).

We find that for the largest percentage of GFC pixels deforested correspond to the associated deforestation year, or the following year (e.g., deforestation classified as deforested in 2003 in GFC may be classified as deforested in PRODES in 2004). To some extent, the classifications of similar pixels across different years is likely a function of the different temporal windows used in the classification methods (PRODES is based on dry season classifications, while GFC is based on the growing season months). The majority of areas classified as deforested in the GFC data, but which are not classified as deforested in the PRODES data, are largely classified as either forest or secondary forests in the PRODES classifications. We note that in Tables S4-S9, the PRODES classification category "1997" refers to areas deforested pre-1997; the category "2001" includes areas deforested between 1997 and 2001.

Patch Size in the GFC data

To investigate the deforestation patch size in the GFC data we used the region group tool in ArcGIS to create clusters of deforestation for each year since 2000. We then estimated the total area deforested in pixels less than less than 6.25 and 25 hectares. 6.25 hectares corresponds to the lowest size threshold for a deforestation classification in PRODES (and is also the equivalent of one Modis pixel) and 25 hectares, the trigger point required to initiate a DETER warning. We include the results as Table S10. In total, we find that an average of 5,866km² of GFC deforestation occurred in areas less than 6.25ha in size. This figure remained relatively from 2002-2013. During this period, an average of 9,828km² were deforested

in clusters less than 25ha. This figure fell slightly over the period 2008 to 2013, from an average of 10,700km² to an average of 8,500km².

Pixel-level Comparison of Cumulative PRODES vs. GFC Forest Loss

We catalogued the overlap of forest cover extent, forest loss, and forest loss patch size in GFC vs. PRODES over the period 2001 to 2013. PRODES and GFC monitor different areas for forest loss and have different reporting thresholds. PRODES reports forest loss patches in excess of 250m, much larger than the 30m minimum reported by the GFC

GFC forest extent and forest cover loss as available as 30m raster data. The legal Amazon falls within 12 GFC granules. Each granule covers is 10 degrees. The GFC forest data is available as raster maps that classify the percentage of forest cover present in the calendar year 2000. We reclassified these granules into a binary map such that a pixel value of 1 indicated pixels with at least 50% forest coverage. All other area was classified as 0. The GFC forest cover loss map is a raster containing values corresponding to the year of forest loss. We reclassified the forest cover loss map into a binary map of forest loss (1) and non-loss (0), where forest cover loss indicates the presence of forest cover loss in any year over the period 2001 to 2013.

The PRODES forest cover and forest cover loss maps are available as 90m raster data. We resampled the PRODES forest cover/loss map to a 30m grid using pixel replication to make it consistent with the GFC dataset. We then reclassified the 30m grid such that any forest loss occurring between 2002 and 2013 was classified as 1. All other classes were reclassified as 2.

A simple overlay of the PRODES and GFC datasets showed that patches of forest and forest loss in PRODES were shifted more than 90m Northeast relative to the GFC (Figure S2). To fix this shift, PRODES images and GFC datasets were co-registered into the same spatial domain using GFC images as the reference dataset. First, the PRODES image was extracted into 12 non-overlapping layers corresponding to the 12 GFC granules. At least 10 geo-reference points representing sharp corners of large deforested and forested patches were used to co-register each PRODES granule with the corresponding GFC granule.

Next each of the 12 non-overlapping PRODES maps was used to derive two separate binary maps representing PRODES forest loss and PRODES forest. In the PRODES forest loss layer, 1 denotes forest loss occurred in period 2002-2013, while 1 in PRODES forest area refers to area that were classified as forest in 2013, classified as forest loss over the period 1995-2001 and were not identified as forest loss in 2002-2013.

Figure S3 shows the five major classes that were used to assess agreement or disagreement between the four binary maps created from the two datasets. Class 1 represents areas that were classified as forest loss by GFC during the period 2001 to 2013 and by PRODES during the period 2002-2013.Class 2 represents the portion of PRODES forest loss during the period 2001-2013 that fell within the GFC forested area but not within the GFC forest loss area for the period 2002-2013. Class 3 is PRODES forest loss during 2002-2013 that was not monitored for forest loss by the GFC. Class 4 represents GFC forest loss during the period 2001-2013 within PRODES forested area that were not reported as forest loss by PRODES in any of the years between 2002 and 2013. Class 5 represents GFC forest loss during 2001-2013 that PRODES did not monitor for forest loss.

The input raster images included four binary maps: GFC forest loss (A), PRODES forest loss (B), GFC forest cover (C), and PRODES forest cover (D). The “Reclassify” tool in ArcGIS (ESRI Inc.,

Redlands, CA) was used to create these binary maps. Raster analysis was performed in the model maker function of ERDAS Imagine (Hexagon Geospatial Inc.) to generate a binary map for each forest loss class (equations (1)(2) - (5)). Note that equations (2) - (5) generate raster maps with values other than 1 and 0 (i.e. 10 and 11). We reclassified these values to 0. The raster analysis was performed at each GFC granule level and then later mosaicked into Amazon Biome and the Brazilian Legal Amazon.

Class 1 binary map was generated using simple intersection (or raster multiplication) of PRODES and GFC forest loss layers as:

$$\text{Class 1} = A \cap B \quad (1)$$

For the Class 2 binary map, binary grid output from intersection of GFC forest and PRODES forest (i.e. 1 = PRODES forest loss in GFC forest cover layers) was added with GFC forest loss layer as:

$$\text{Class 2} = \text{Con}(B \cap C + 10 \times A = = 1,1,0) \quad (2)$$

The Class 3 binary map was created using simple raster addition as:

$$\text{Class 3} = \text{Con}(B + 10 \times C = = 1,1,0) \quad (3)$$

For the Class 4 binary map, binary grid output from intersection of GFC forest loss and PRODES forest cover layers was added with PRODES forest loss layer as:

$$\text{Class 4} = \text{Con}(A \cap D + 10 \times B = = 1,1,0) \quad (4)$$

Class 5 binary map was created by adding GFC forest cover layer with union of PRODES forest cover and PRODES forest loss layers as:

$$\text{Class 5} = \text{Con}(A + 10 \times (B \cup D) = = 1,1,0) \quad (5)$$

Table S13 and S14 list area and percent coverage of different forest loss classes in the Amazon Biome and Legal Amazon, respectively. Around 73% of forest loss in the Amazon biome (or 72% in the legal Amazon) of total forest loss reported by PRODES was also identified as forest loss by GFC datasets. However, only 51% of total GFC forest loss (2001-2013) in Amazon biome (or 45% in the legal Amazon) was reported in PRODES during the period 2002-2013.

Class 4 is of particular interest as it measures area of GFC forest loss during 2001-2013 that was not reported by PRODES in the period 2002-2013 and because GFC measure much more forest loss than PRODES. Roughly 49% and 55% of total GFC forest loss area in the Amazon Biome and the Legal Amazon, respectively, were not reported by PRODES.

We found that little of this difference is explained by the difference in the PRODES vs. GFC minimum detectable unit. We reclassified the Class 4 raster into 250 m using a majority filter. The results Table S15 shows that the designation of minimum reporting unit in PRODES accounted for 26% of the mismatch PRODES omission from GFC in the Legal Amazon and 28% in the Amazon Biome.

Medium-Scale Analysis

To better identify regional differences between the GFC and PRODES data create a regional map of the differences between the GFC and PRODES classifications. To accomplish this, we first created a series of 30km x 30km grid cells over the entire legal Amazon by aggregating the 30m raster pixels into 1,000 pixel squares. This resulted in a total of 30km x 30km ($900km^2$) grid cells. We then estimated the total area deforested in both the GFC and PRODES data within each grid cell, as well as the number of fire incidents and total burned area.

Spatial Distribution of GFC and PRODES deforestation

In Figure S4 we map total deforestation per grid cell between 2009 and 2013 for both the GFC (top) and PRODES (bottom) classifications. Both the GFC and PRODES classifications identify deforestation hotspots in southwest and eastern Pará state (the latter area is referred to locally, as the "South of Pará), and in western Rondônia. The two datasets do differ, however, in several key areas. First, the GFC data identifies deforestation along the principal channel of the Amazon River. Second, the GFC classifies significant deforestation in Mato Grosso, and in particular in areas around the soybean producing region in the north-central portion of the state. The difference in deforestation recorded in Mato Grosso by PRODES and GFC is particularly striking after 2008 (see Figure S4).

Correlations between GFC, PRODES and MDC45A1 (Burned Area Product)

For each aggregated 30x30 km grid with non-zero deforestation or non-zero burned area, we calculated the contemporaneous linear correlation of both deforestation datasets (GFC and PRODES) with burned area (MDC45A1) between the years 2002-2013 and plotted a histogram of the corresponding correlation values (Figure S5). GFC and burned area (BA1) are more positively correlated with each other (dark red) than PRODES and BA1. Areas with high positive correlation include areas with active deforestation such as São Félix do Xingu, southwest Pará along BR-163, and northern Mato Grosso. The average correlation of GFC/BA1 and PRODES/BA1 is 0.255 and 0.138 respectively. The histogram shows that positive correlation values for GFC/BA1 (blue bars) are more frequent than the corresponding PRODES/BA1 values (magenta bars).

Deforestation Trends

We calculated trends in deforestation using both GFC and PRODES datasets for the periods between 2002-2013 and 2009-2013. Figure S6 reports the t-statistics of the time coefficient β_1^i for each 30x30 km grid *i* according to the following regression:

$$d_t^i = \beta_0^i + \beta_1^i t + \mu_t^i \qquad (6)$$

where d is the deforested area. We emphasize that Figure S6 displays the trend results for grids with accumulated deforestation above 900 ha (or 1% of the grid area) between 2002-2013 and between 2009-2013.

Deforestation trends are negative (blue cells) for both PRODES and GFC between 2002-2013 for large portions of Rondônia, Mato Grosso, and southern Pará. This negative trend could be due to anti-deforestation policies and supply-chain mechanisms or because deforestation is already close to the physical upper bound limit of the grid. The mean estimated coefficient of deforestation trend between 2002-2013 for the PRODES data is -119.5 and the largest is 260.75. For the GFC dataset, the average trend coefficient is -62.38 with a maximum value of 303.34. These descriptive statistics are only for the grids with accumulated deforestation greater than 900 ha. For the period 2009-2013, the t-statistic values are smaller in magnitude. These smaller values are likely to be due to the shorter four year period, which impacts not only the magnitude of trend coefficient but also the standard error.

Differences in Differences Analysis

Differences in differences (DD) is a statistical method used to estimate causal relationships. The technique examines a dependent variable summarizing a characteristic of a treated sub-sample before and

after a treatment relative to a non-treated subsample before and after the treatment (*12*). A typical use of the approach might compare, for example, business activity in states where a regulation has been changed relative to business activity in states where that same regulation has been left unchanged. To estimate the effect of the treatment, the technique is to perform a regression of the general form shown in this equation:

$$y_{it} = B_0 T + B_1 P + B_2(T * P) + \boldsymbol{B_3 C} + \qquad (7)$$

The regression controls for whether each observation has been exposed the treatment, *T*, whether it occurs in the pre-treatment period vs. the post-treatment period, *P*, the interaction between presence in the treated group during the post treated period, *T**P* and a vector of other controls deemed relevant, *C*. The interaction term gives the causal effect of the treatment. This term represents the difference in the observed post-treatment values of the dependent variable from a counterfactual prediction of the values of the dependent variable had no treatment occurred.

We hypothesize that the commencement of the use of PRODES as not only a monitoring, but also an enforcement tool, caused a portion of the decline in deforestation reported by the PRODES system in recent years. If this were true, we would expect that PRODES deforestation rates across the biome would decline after 2008 relative to the decline reported by the GFC or by any monitoring system left unbiased (untreated) by the advent of PRODES-based enforcement. Thus we class PRODES deforestation reports in each pixel as treated and class GFC deforestation reports as untreated. The treatment period, *P*, is assigned the value of zero for the period 2002 to 2008 and 1 for 2009 to 2013. To ensure that the effect is not just an artifact of the difference in monitoring calendars of the two systems, we performed the analysis on moving averages of sets of three years. Thus, the year 2002 in the dataset contains values from period January 2001 to December 2003 for GFC and August 2001 to July 2004 for PRODES. The analysis was performed at the level of 30km grid cells (n=4,931). The results reveal a significant negative effect on PRODES deforestation in years following 2008 ($p<0.01$).

Figure S7 depicts the predicted values from regression C. It shows the difference between PRODES, GFC, and the post-2008 PRODES counterfactual with the same trend in reduced deforestation as the GFC. The wedge between the PRODES counterfactual line and the blue line is the estimated amount that enforcement caused PRODES to underestimate deforestation. The area of this wedge is roughly 900,000 hectares, an area roughly the size of the island of Puerto Rico. Table S13 contains the regression results and demonstrates that the treatment effect is robustly negative across several specifications.

Estimates of Carbon Emissions

We estimate carbon emissions from deforestation since 2008 based on two widely cited measures of above ground live biomass: the Amazon Basin Aboveground Live Biomass (ABALB) distribution map (*13*) and the Pantropical National Level Carbon Stock (PNLCS) dataset (*14*). The former was created specifically for the Amazon, and is based on a combination of land cover maps, remote sensing, and field measurements from the period 1990-2000, and classifies biomass according to one of twelve levels ranging from 0 to more than 400tons of biomass per ha, with classification levels increasing in 25 or 50mg/ha increments. The ABALB data, along with PRODES, has also been used as the basis for estimating emissions from tropical deforestation in the Amazon (*15*). The PNLCS dataset is based on a

combination of field measurements, LiDAR, and 2007-2008 MODIS imagery. Unlike the ABALB data, the PNLCS dataset covers the extent of the tropics. The ABLB data is also produced in 1km pixels. The PNLCS data is available at a slightly finer resolution, at 500m pixels.

The later imagery used in the PNLCS dataset is advantageous to our analysis. Given that we expect that the GFC data is capturing emissions from the deforestation of both secondary and primary forests, the PNLCS would provide a more accurate estimate of carbon in regrown areas. The PLCS data is also continuous, while the ABALB data classified biomass levels into one of twelve categories. Given the importance of the ABALB data to Brazil's public policy and emissions reductions programs, however, and that the dataset was created specifically for measuring above ground live biomass in the Amazon Basin, the AGALB data also possesses several advantages to our analysis. Rather than choose one dataset for our analysis we estimated carbon emissions using both datasets. .

To estimate carbon emissions we began by projecting both the ABALB and PNLCS data into an equal area projection and then resampling the results into 30m pixels (using the nearest neighbor classification method). Next, we weighted each deforested pixel according to the estimated AGLB (above ground live biomass) values for each year. Third, we converted the total number of pixels per AGLB weight to hectares (each 30m² pixel equals 0.09ha), then multiplied the result by the estimated carbon levels (e.g., AGLB *0.5). We note that for the ABALB dataset we weighted pixels according to the middle value for each category (e.g., all pixels estimated as having between 25 and 50mg/ha of biomass were classified as having 37.5mg/ha). For the PNLCS data, which provides continuous estimates, we actual value associated with the specific pixel. Finally, we summed the weighted values for each year from 2002-2013 to produce our estimates of emitted carbon. We include the full results as Tables S11-S12.

When using the PNLCS estimates of carbon stocks and the GFC deforestation data, we estimate that a total of 494Tg of carbon were lost from deforestation in the Amazon Biome during the 2009-2013 period. When using the PNLCS data and the PRODES indicators for forest loss, we estimate that only 242Tg of carbon were lost due to forest clearing. We also find, however, the average levels of carbon lost per hectare were significantly higher when using the PRODES data than the GFC figures. For example, we estimate an average of approximately 82.5t/ha of carbon emitted when our estimates are based on the GFC data, as opposed to an average of 93.16t/ha when using the PRODES indicators. This difference is expected, however, given that the GFC data includes deforestation in the drier, scrub forests within the biome, and in areas of secondary forests.

Estimates of carbon emissions based on ABALB data correspond closely with the estimates based on the PNLCS data. When using the ABALB carbon estimates and the GFC deforestation indicators, we estimate that total carbon emissions from deforestation in the Amazon equated to approximately 511Tg, with an average of 84.8t of carbon emitted per hectare. When using the PRODES data we again find that estimates are significantly less, at approximately 256Tg of carbon, emitted at a rate of approximately 97.9t/ha.

SOURCES

1. A. B. Rylands, K. Brandon, Unidades de conservação brasileiras. *Megadiversidade* **1**, 27-35 (2005).
2. Brazil, M. d. M. Ambiente, Ed. (Brasilia, Brazil, 2013).
3. B. S. Soares-Filho *et al.*, Modelling conservation in the Amazon basin. *Nature* **440**, 520-523 (2006).
4. E. Y. Arima, P. Barreto, E. Araújo, B. Soares-Filho, Public policies can reduce tropical deforestation: Lessons and challenges from Brazil. *Land Use Policy* **41**, 465-473 (2014).
5. Ministerio Publico Federal. (http://www.prpa.mpf.mp.br/news/2009/outros/Apresentacao%20TAC%20Bois%20Desmatamento.ppt/view, 2009).
6. INPE. (National Institute of Space Research, http://www.dpi.inpe.br/prodesdigital/dadosn/, 2015), vol. 2015.
7. M. C. Hansen *et al.*, High-Resolution Global Maps of 21st-Century Forest Cover Change. *Science* **342**, 850-853 (2013).
8. N. FIRMS. (NASA FIRMS, Available on-line: [https://earthdata.nasa.gov/active-fire-data]. 2015 MODIS Active Fire Detections extracted from MCD14ML).
9. L. Boschetti, A. D. Roy, A. A. Hoffman, M. Humber, "MODIS Collection 5.1 Burned Area Product - MCD45 Version 3.0.1," (2013).
10. INPE, "Methodology for Calculating Annaul Deforestation Rates in the Legal Amazon [Metodologia para o Cálculo da Taxa Anual de Desmatamento na Amazônia Legal]," (Instituto Nacional de Pesquisas Espaciais Coordenadoria Geral de Observação da Terra Programa Amazônia – Projeto PRODES São Jose dos Campos, SP, Brazil http://www.obt.inpe.br/prodes/metodologia_TaxaProdes.pdf, 2013).
11. R. Tropek *et al.*, Comment on “High-resolution global maps of 21st-century forest cover change”. *Science* **344**, 981-981 (2014).
12. J. D. Angrist, J.-S. Pischke, *Mostly harmless econometrics: An empiricist's companion*. (Princeton university press, 2008).
13. S. S. Saatchi, R. A. Houghton, D. Alves, B. Nelson. (Oak Ridge National Laboratory Distributed Active Archive Center, Oak Ridge, Tennessee, 2009).
14. A. Baccini *et al.*, Estimated carbon dioxide emissions from tropical deforestation improved by carbon-density maps. *Nature Clim. Change* **2**, 182-185 (2012).
15. D. Nepstad *et al.*, Slowing Amazon deforestation through public policy and interventions in beef and soy supply chains. *Science* **344**, 1118-1123 (2014).

Figure S1

Deforestation and Fire Incidents Pre and Post-2008

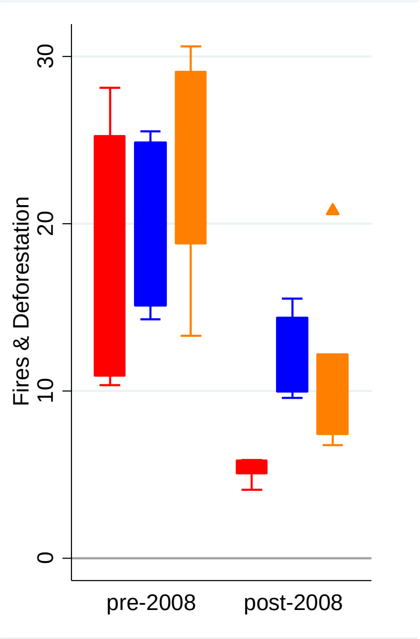

Distribution of deforestation (in 1000s km²) observed in the PRODES (red) and GFC (blue) and number of FIRMS fire incidents (orange, in 10,000 units) per year, pre and post-2008. By all measures, deforestation and land clearing activity declined from the first to the second period. Declines are substantially smaller in the GFC data than in the PRODES data. Pre-2008, the GFC and PRODES distributions are closely correlated. After 2008, however, the PRODES and GFC data diverge, and the FIRMS fire incidents maintain their correlation with only the GFC data.

Figure S2

Amazon Biome forest loss maps from GFC and PRODES for the years 2001-2013 and 2002-2013, respectively. The small image shows the shift in dataset before co-registration. The small image at right shows the revised position of PRODES pixels after co-registration. Co-registration increased the area of intersection of PRODES and GFC forest loss by about 8% in the Legal Amazon.

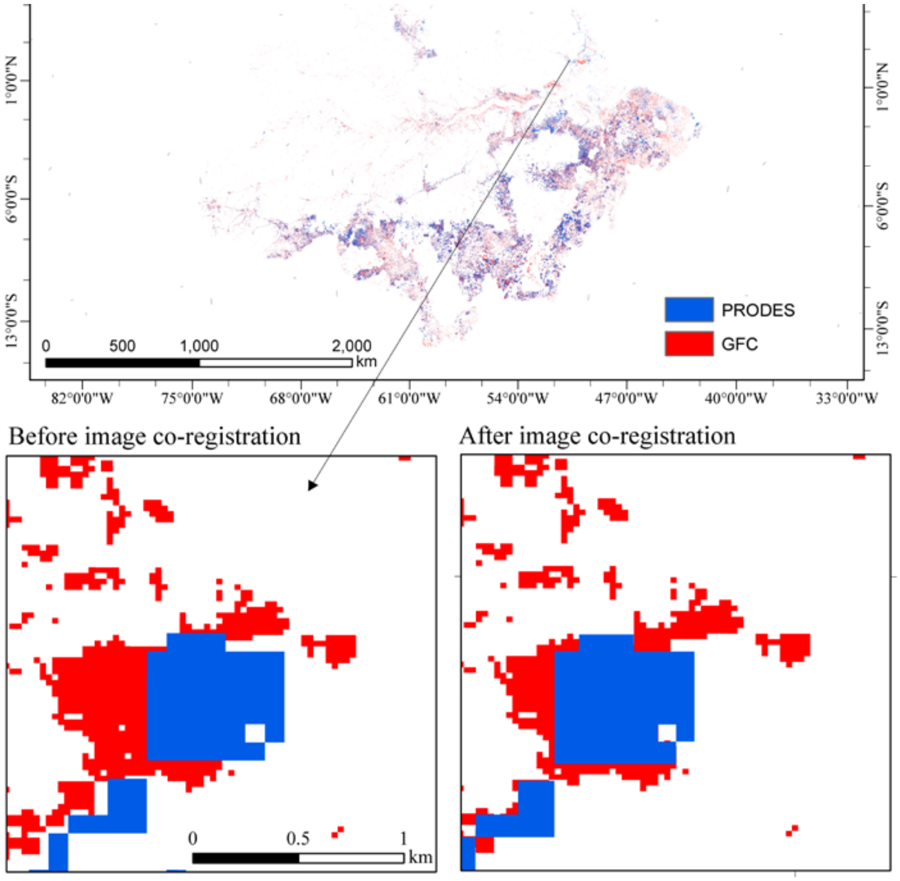

Figure S3

Five major classes used to study the agreement (or disagreement) between GFC and PRODES in terms of identifying the spatial extent of forest loss in the Amazon Biome. The size of the shapes are proportional to the areas of each class.

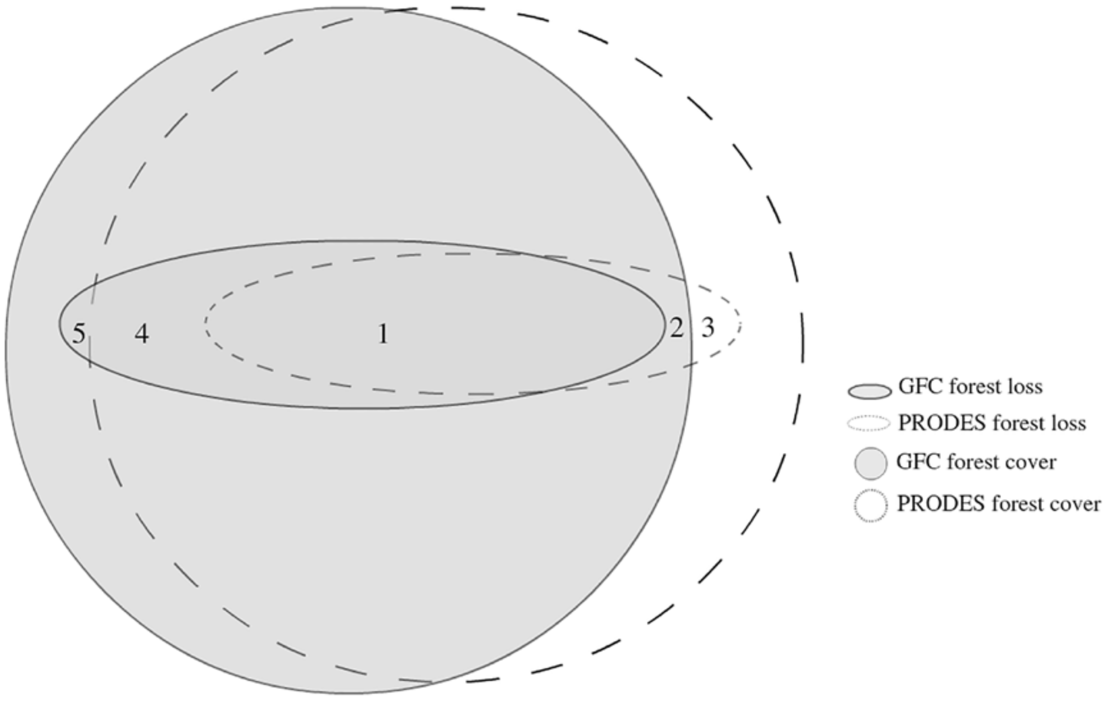

Figure S4
Total Deforestation per 900km² Aggregated Pixel Areas, 2009-2013.

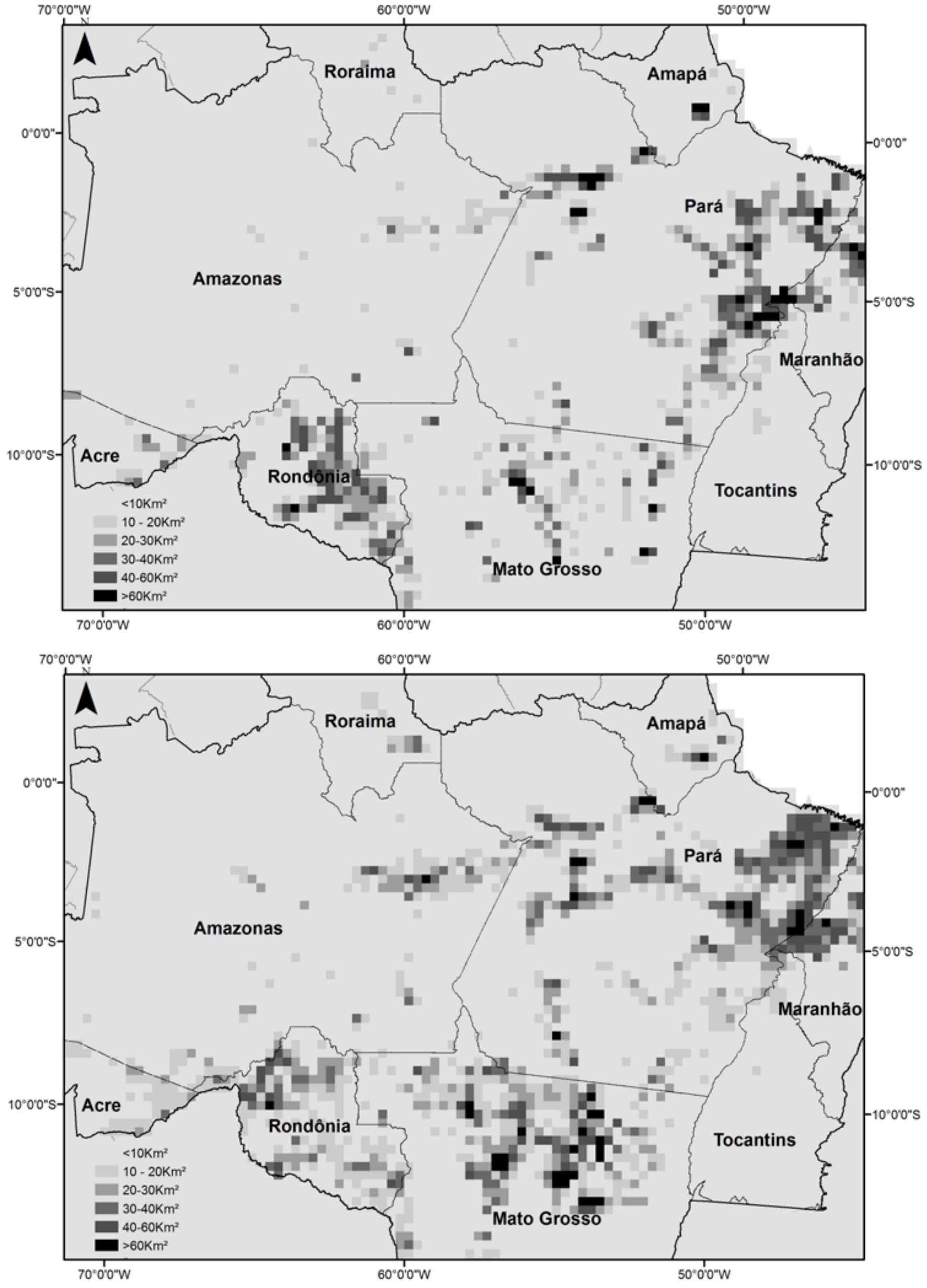

Differences in total deforestation per 900km² pixel, as classified according to GFC and PRODES, over the periods 2002-2008 (top) and 2009-2013 (bottom). Total losses ranged from 0 to 220km² per 900km² pixel. The largest differences between the two products after 2008 are found in north-central Mato Grosso and in areas along the main trunk of the Amazon River.

Figure S5
Correlation between deforestation datasets and burned area

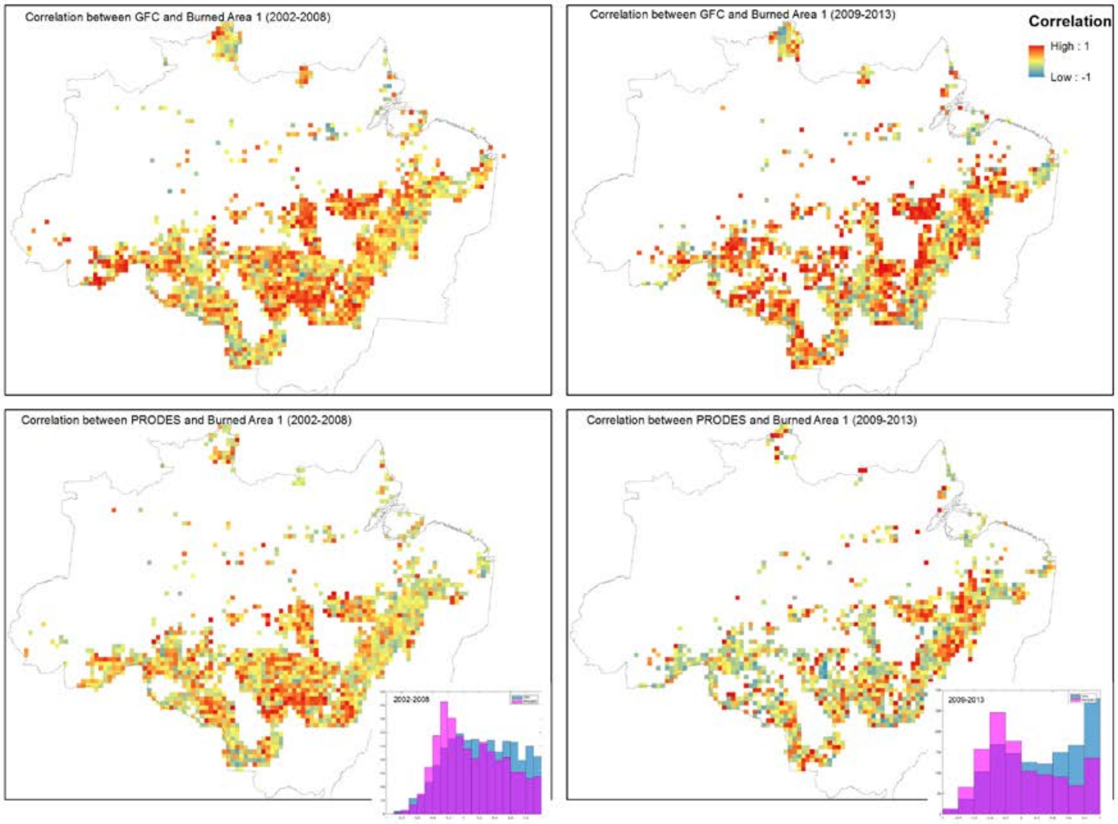

Comparisons of GFC, PRODES and burned area per each 30km x 30km grid cell suggest that GFC is more closely correlated with the burned areas in the Amazon Biome, particularly since 2008. Figure S5 maps the correlation between both PRODES and GFC with Burned Areas during the pre and post 2008 periods. Darker red areas indicate positive correlation. Pre 2008 (left), both the GFC and PRODES data are closely correlated with the burned area data. However, after 2008 (right panels), the correlation between the PRODES and burned area declines markedly. The histograms at the bottom right of each column show that positive correlation values for GFC/BA1 (blue bars) are more frequent than the corresponding PRODES/BA1 values (magenta bars).

Figure S6
Deforestation Trends

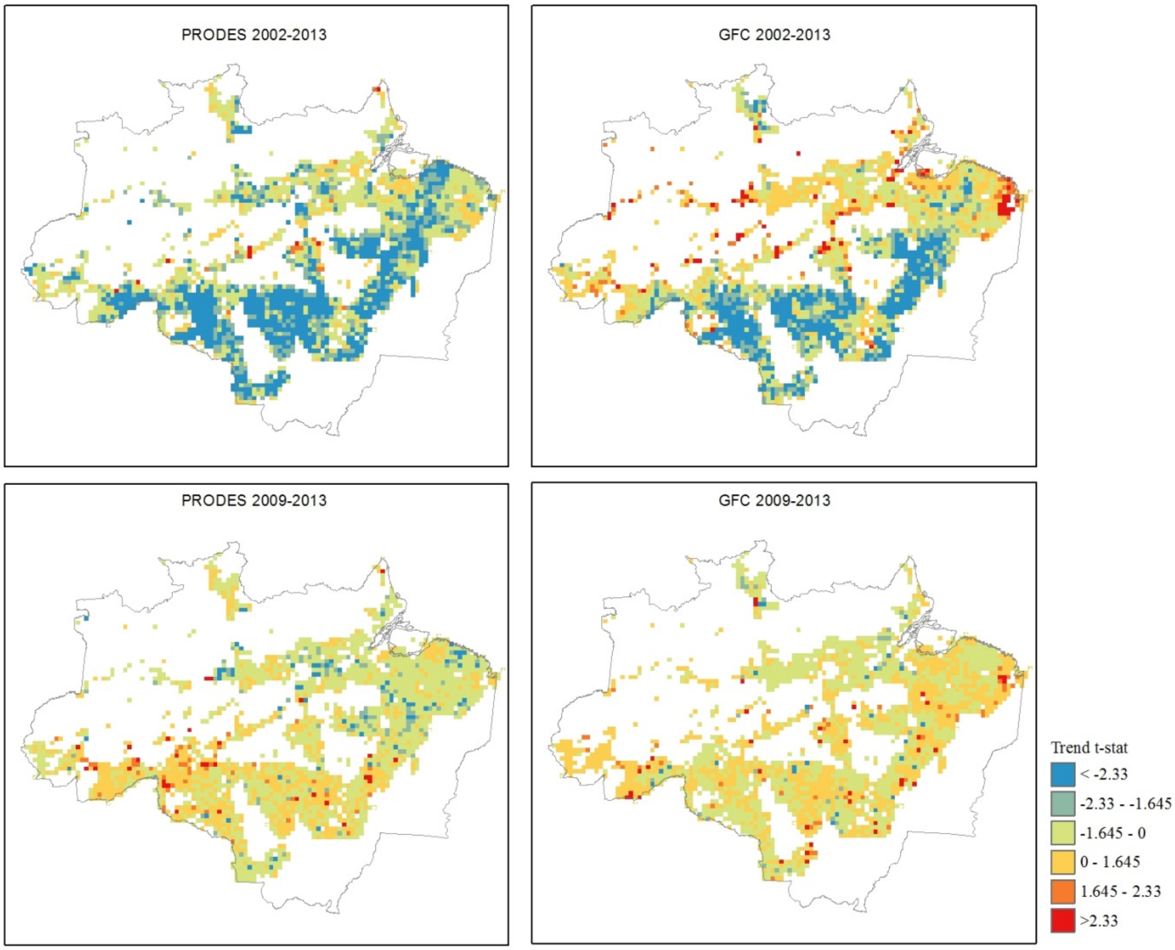

Figure S6 maps t-statistics associated with a linear estimate of forest loss at the grid cell scale, for both the PRODES and GFC data. Trends are negative (blue cells) for both PRODES and GFC between 2002-2013 for large portions of Rondônia, Mato Grosso, and southern Pará. For the period 2009-2013, the t-statistic values are smaller in magnitude. Each map displays the trend results for grids with accumulated deforestation above 900 ha (or 1% of the grid area) between 2002-2013, or between 2009-2013.

Figure S7

The estimated effect of the use of PRODES as an enforcement tool on PRODES deforestation rates over the period 2008 – 2012.

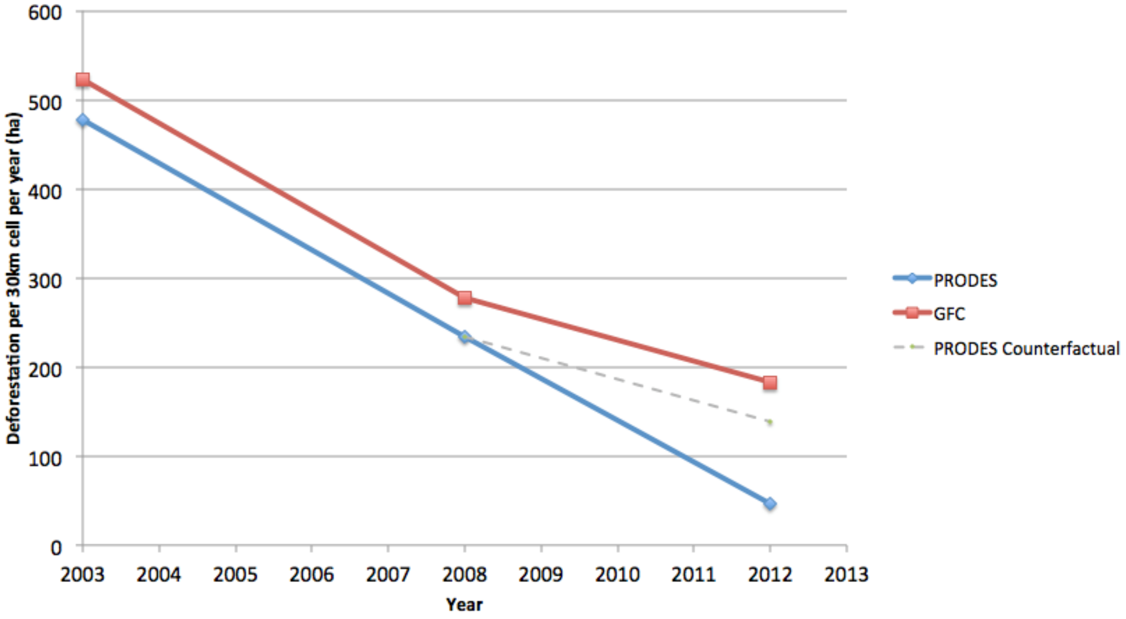

Values depicted for the GFC and PRODES are predicted values obtained from regression C. Values for the PRODES counterfactual are obtained by shifting the intercept of the 2008 -2012 segment of the GFC data. The results reveal an estimated discrepancy of greater than 900,000 hectares over the period.

Table S1.

Deforestation (km²) and Fire Incidents in the Amazon Biome, 2002-2013

Year	PRODES	GFC	FIRMS	
2002	23,763	23,387	214,726	
2003	28,137	20,418	228,166	
2004	25,241	25,519	290,827	
2005	21,923	24,853	306,051	
2006	10,350	18,726	188,218	
2007	10,921	15,072	266,577	
2008	12,351	14,292	133,150	PPCDAm ii enacted
2009	5,887	9,959	102,483	
2010	5,845	15,517	207,803	
2011	5,238	10,816	74,100	
2012	4,111	14,390	122,027	
2013	5,075	9,584	67,875	

Table S2
Correlation Matrix

2002-2008	PRODES	GFC
GFC	0.77*	
FIRMS	0.45	0.65

2009-2013	PRODES	GFC
GFC	-0.19	
FIRMS	0.29	0.87*

**indicates significance 0.1 levels*

Table S3
T tests

	n	mean	SD	t	df	p
2002-2008						
PRODES	7	18,955	4,535			
GFC	7	20,323	7,505			
Diff		1,369	4,940	0.73	6	0.4912
2009-2013						
PRODES	5	5,231	322			
GFC	5	12,053	2,714			
Diff		6,822	1,314	5.19	4	0.0066

Table S4
GFC PRODES Gross Totals, by km2

Year	2002	2003	2004	2005	2006	2007	2008	2009	2010	2011	2012	2013
No obs	23	13	29	28	27	13	36	28	29	20	25	17
Forest	2,244	2,130	3,074	3,219	2,761	2,562	2,606	1,969	3,837	2,668	4,337	3,011
Cloud	178	150	259	241	253	252	287	392	377	268	519	498
Water	63	45	49	89	72	47	52	100	89	71	120	46
Non-For	798	871	919	630	601	564	523	407	689	500	702	361
Pre1997	5,383	3,959	4,239	4,743	3,404	2,737	2,572	2,191	2,956	2,167	2,405	2,585
2000	1,772	1,178	1,287	1,493	898	575	684	398	627	485	575	411
2001	1,143	800	911	893	697	528	563	371	508	370	411	415
2002	6,884	798	1,239	1,121	645	379	467	199	421	285	302	123
2003	2,524	6,613	1,339	1,759	817	548	641	360	535	369	436	147
2004	880	2,056	7,707	1,578	1,173	691	722	412	621	404	414	139
2005	653	891	2,604	6,570	1,097	838	768	398	639	381	393	106
2006	175	217	586	946	3,419	408	457	253	353	211	214	61
2007	174	187	406	488	1,468	2,881	524	380	510	299	280	80
2008	211	230	385	422	717	1,291	2,077	402	728	410	434	127
2009	76	78	129	176	218	257	615	898	377	233	304	83
2010	72	66	117	147	160	159	296	427	1,061	211	272	90
2011	55	59	115	134	139	146	188	197	693	851	323	99
2012	36	33	56	81	72	92	103	85	240	384	1,101	129
2013	41	42	64	94	88	102	110	92	225	230	823	1,055
	23,385	20,418	25,516	24,853	18,725	15,071	14,290	9,958	15,516	10,816	14,389	9,582

Classification of GFC deforestation incidents (by year in columns) in PRODES. Corresponding years are highlighted. Areas deforested prior to 1997 are in bold. Areas are in square kilometers.

Table S5
GFC PRODES Percent Deforested

Year	2002	2003	2004	2005	2006	2007	2008	2009	2010	2011	2012	2013
No obs	0%	0%	0%	0%	0%	0%	0%	0%	0%	0%	0%	0%
Forest	10%	10%	12%	13%	15%	17%	18%	20%	25%	25%	30%	31%
Cloud	1%	1%	1%	1%	1%	2%	2%	4%	2%	2%	4%	5%
Water	0%	0%	0%	0%	0%	0%	0%	1%	1%	1%	1%	0%
Non-For	3%	4%	4%	3%	3%	4%	4%	4%	4%	5%	5%	4%
Pre-1997	23%	19%	17%	19%	18%	18%	18%	22%	19%	20%	17%	27%
2000	8%	6%	5%	6%	5%	4%	5%	4%	4%	4%	4%	4%
2001	5%	4%	4%	4%	4%	4%	4%	4%	3%	3%	3%	4%
2002	29%	4%	5%	5%	3%	3%	3%	2%	3%	3%	2%	1%
2003	11%	32%	5%	7%	4%	4%	4%	4%	3%	3%	3%	2%
2004	4%	10%	30%	6%	6%	5%	5%	4%	4%	4%	3%	1%
2005	3%	4%	10%	26%	6%	6%	5%	4%	4%	4%	3%	1%
2006	1%	1%	2%	4%	18%	3%	3%	3%	2%	2%	1%	1%
2007	1%	1%	2%	2%	8%	19%	4%	4%	3%	3%	2%	1%
2008	1%	1%	2%	2%	4%	9%	15%	4%	5%	4%	3%	1%
2009	0%	0%	1%	1%	1%	2%	4%	9%	2%	2%	2%	1%
2010	0%	0%	0%	1%	1%	1%	2%	4%	7%	2%	2%	1%
2011	0%	0%	0%	1%	1%	1%	1%	2%	4%	8%	2%	1%
2012	0%	0%	0%	0%	0%	1%	1%	1%	2%	4%	8%	1%
2013	0%	0%	0%	0%	0%	1%	1%	1%	1%	2%	6%	11%

Percentage of GFC deforestation incidents per year (in columns) classified according to each PRODES category. Corresponding years are highlighted. Areas deforested prior to 1997 are in bold.

Table S6

FIRMS PRODES Gross Fire Totals

Year	2002	2003	2004	2005	2006	2007	2008	2009	2010	2011	2012	2013
No obs	418	317	470	455	293	334	315	259	436	217	257	133
Forest	31,008	39,229	51,344	60,890	39,423	62,084	28,572	20,515	59,350	15,549	30,557	15,664
Cloud	3,302	4,155	4,377	3,946	3,550	3,895	2,876	4,651	2,957	2,065	3,496	2,394
Water	2,049	1,828	1,838	2,193	1,787	1,809	1,461	1,706	2,193	1,365	1,499	1,107
Non-For	17,156	18,065	23,741	18,688	12,317	20,005	12,009	10,582	21,848	10,563	14,817	7,758
Pre1997	61,147	54,287	59,672	61,116	35,765	54,260	27,108	24,037	38,322	17,194	24,286	16,963
2000	25,609	18,999	19,759	20,708	9,871	15,059	6,468	4,747	9,507	3,173	5,139	3,127
2001	17,826	13,617	12,629	13,261	6,833	9,412	5,217	4,378	6,240	2,902	3,952	2,499
2002	27,451	19,907	20,574	16,722	7,763	10,456	4,434	2,587	6,354	1,852	2,927	1,631
2003	10,717	32,170	30,793	25,812	9,946	13,189	5,538	3,702	7,494	2,587	3,746	2,354
2004	5,692	10,721	37,452	29,865	18,084	17,663	7,843	4,907	9,369	3,259	4,578	2,726
2005	4,476	5,243	12,934	31,183	16,489	21,254	8,131	4,773	9,894	2,786	4,770	2,802
2006	1,507	1,822	3,330	5,473	11,443	8,290	5,677	2,624	5,243	1,260	2,352	1,124
2007	1,493	1,828	3,273	4,123	5,602	11,587	5,350	3,635	6,960	1,715	2,995	1,108
2008	1,963	2,475	3,490	4,245	3,576	8,698	5,904	3,000	7,746	1,848	3,517	1,404
2009	727	829	1,187	1,764	1,442	2,092	2,308	2,813	3,246	1,392	2,356	655
2010	641	783	1,161	1,678	1,125	1,750	1,229	1,517	3,938	1,149	2,057	771
2011	599	756	1,105	1,520	1,147	1,782	1,047	912	3,048	1,672	2,845	979
2012	441	486	758	1,149	794	1,464	730	513	1,764	861	3,297	821
2013	504	649	940	1,260	968	1,494	933	625	1,894	691	2,584	1,855
Total	214,726	228,166	290,827	306,051	188,218	266,577	133,150	102,483	207,803	74,100	122,027	67,875

Number of FIRMS fire incidents (by year in columns) in PRODES. Corresponding years are highlighted. Areas deforested prior to 1997 are in bold.

Table S7

FIRMS PRODES- Percent

Year	2002	2003	2004	2005	2006	2007	2008	2009	2010	2011	2012	2013
No obs	0%	0%	0%	0%	0%	0%	0%	0%	0%	0%	0%	0%
Forest	14%	17%	18%	20%	21%	23%	21%	20%	29%	21%	25%	23%
Cloud	2%	2%	2%	1%	2%	1%	2%	5%	1%	3%	3%	4%
Water	1%	1%	1%	1%	1%	1%	1%	2%	1%	2%	1%	2%
Non-For	8%	8%	8%	6%	7%	8%	9%	10%	11%	14%	12%	11%
Pre1997	28%	24%	21%	20%	19%	20%	20%	23%	18%	23%	20%	25%
2000	12%	8%	7%	7%	5%	6%	5%	5%	5%	4%	4%	5%
2001	8%	6%	4%	4%	4%	4%	4%	4%	3%	4%	3%	4%
2002	13%	9%	7%	5%	4%	4%	3%	3%	3%	2%	2%	2%
2003	5%	14%	11%	8%	5%	5%	4%	4%	4%	3%	3%	3%
2004	3%	5%	13%	10%	10%	7%	6%	5%	5%	4%	4%	4%
2005	2%	2%	4%	10%	9%	8%	6%	5%	5%	4%	4%	4%
2006	1%	1%	1%	2%	6%	3%	4%	3%	3%	2%	2%	2%
2007	1%	1%	1%	1%	3%	4%	4%	4%	3%	2%	2%	2%
2008	1%	1%	1%	1%	2%	3%	4%	3%	4%	2%	3%	2%
2009	0%	0%	0%	1%	1%	1%	2%	3%	2%	2%	2%	1%
2010	0%	0%	0%	1%	1%	1%	1%	1%	2%	2%	2%	1%
2011	0%	0%	0%	0%	1%	1%	1%	1%	1%	2%	2%	1%
2012	0%	0%	0%	0%	0%	1%	1%	1%	1%	1%	3%	1%
2013	0%	0%	0%	0%	1%	1%	1%	1%	1%	1%	2%	3%

Percentage of FIRMS fire incidents per year (in columns) classified according to each PRODES category. Corresponding years are highlighted. Areas deforested prior to 1997 are in bold.

Table S8
FIRMS GFC Gross Fire Totals

Year	2002	2003	2004	2005	2006	2007	2008	2009	2010	2011	2012	2013
Forest	135,228	126,442	148,733	158,082	95,989	149,513	74,803	63,381	126,627	47,363	73,394	43,713
2001	12,073	10,544	7,834	8,276	3,772	4,874	2,230	1,715	3,064	1,074	1,620	1,016
2002	30,865	21,187	21,357	15,218	6,959	9,541	3,917	2,415	5,411	1,729	2,434	1,577
2003	5,809	31,395	27,394	21,081	8,059	10,608	4,030	2,719	6,219	1,899	2,761	1,967
2004	6,808	8,717	42,007	28,180	17,679	16,697	7,063	4,338	8,553	2,732	4,078	2,782
2005	5,864	7,852	10,526	34,319	14,781	18,760	7,084	4,289	8,812	2,593	3,920	2,163
2006	3,752	4,575	7,510	9,935	18,673	12,560	7,689	3,728	7,815	1,955	3,406	1,706
2007	2,767	3,295	5,043	6,025	4,656	16,974	5,518	3,844	7,902	1,974	3,313	1,425
2008	2,643	3,294	4,872	6,221	4,263	6,662	10,055	3,454	6,574	1,678	3,125	1,384
2009	1,522	1,942	2,837	3,171	2,715	3,378	2,244	5,600	3,543	1,871	2,501	1,089
2010	2,534	3,139	4,749	5,931	3,995	6,871	3,163	2,706	13,477	2,271	5,466	1,877
2011	1,852	2,202	3,220	3,687	2,585	4,129	1,959	1,431	3,915	4,220	3,550	1,573
2012	1,938	2,485	3,290	4,248	2,843	4,195	2,391	1,901	4,503	1,981	10,605	2,466
2013	1,071	1,097	1,455	1,677	1,249	1,815	1,004	962	1,388	760	1,854	3,137
Total	214,726	228,166	290,827	306,051	188,218	266,577	133,150	102,483	207,803	74,100	122,027	67,875

Number of FIRMS fire incidents (by year in columns) as classified in GFC. Corresponding years are highlighted. Forest areas are shown in green.

Table S9

FIRMS GFC- Percent

Year	2002	2003	2004	2005	2006	2007	2008	2009	2010	2011	2012	2013
Forest	63%	55%	51%	52%	51%	56%	56%	62%	61%	64%	60%	64%
2001	6%	5%	3%	3%	2%	2%	2%	2%	1%	1%	1%	1%
2002	14%	9%	7%	5%	4%	4%	3%	2%	3%	2%	2%	2%
2003	3%	14%	9%	7%	4%	4%	3%	3%	3%	3%	2%	3%
2004	3%	4%	14%	9%	9%	6%	5%	4%	4%	4%	3%	4%
2005	3%	3%	4%	11%	8%	7%	5%	4%	4%	3%	3%	3%
2006	2%	2%	3%	3%	10%	5%	6%	4%	4%	3%	3%	3%
2007	1%	1%	2%	2%	2%	6%	4%	4%	4%	3%	3%	2%
2008	1%	1%	2%	2%	2%	2%	8%	3%	3%	2%	3%	2%
2009	1%	1%	1%	1%	1%	1%	2%	5%	2%	3%	2%	2%
2010	1%	1%	2%	2%	2%	3%	2%	3%	6%	3%	4%	3%
2011	1%	1%	1%	1%	1%	2%	1%	1%	2%	6%	3%	2%
2012	1%	1%	1%	1%	2%	2%	2%	2%	2%	3%	9%	4%
2013	0%	0%	1%	1%	1%	1%	1%	1%	1%	1%	2%	5%

Percentage of FIRMS fire incidents per year (in columns) as classified in the GFC data.
Corresponding years are highlighted. Forest areas are shown in green.

Table S10
GFC Deforestation in Small Plots, 2002-2010
Figures in square kilometers

Year	Total Deforestation in Clusters less than 6.25ha	Total Deforestation in Clusters less than 25ha
2002	6,037	10,699
2003	5,114	9,238
2004	6,129	11,273
2005	7,315	13,427
2006	6,240	11,034
2007	5,584	9,469
2008	5,940	9,814
2009	5,083	7,744
2010	6,782	10,645
2011	5,120	7,936
2012	5,811	9,323
2013	5,232	7,337
Total	70,388	117,938
Average 2002-2008	6,052	10,708
Average 2009-2013	5,605	8,597

Table S11

Estimated Emissions based on GFC and PRODES deforestation, 2002-2013, using the PNLCS dataset.

Year	GFC Total C Emitted (tons)	Area Deforested (ha)	Average C emitted/ha	PRODES Total C Emitted (tons)	Area Deforested (ha)	Average C emitted/ha
2002	114,947,792	23,387	49.15	133,463,623	2,350,726	56.78
2003	101,727,123	20,418	49.82	169,424,381	2,794,927	60.62
2004	137,691,221	25,519	53.96	151,237,979	2,513,517	60.17
2005	144,255,430	24,853	58.04	129,424,181	2,184,370	59.25
2006	116,622,551	18,726	62.28	68,383,213	1,031,561	66.29
2007	103,510,751	15,072	68.68	80,823,557	1,088,694	74.24
2008	105,600,691	14,292	73.89	96,519,070	1,229,087	78.53
2009	80,192,007	9,959	80.52	52,262,058	585,224	89.30
2010	122,203,116	15,517	78.75	52,496,444	577,945	90.83
2011	85,924,295	10,816	79.44	48,518,558	520,858	93.15
2012	122,478,688	14,178	86.39	39,667,030	409,336	96.91
2013	83,904,170	9,492	88.40	49,183,756	505,827	97.23
Total	1,319,057,835	202,229	65.23	1,071,403,850	15,792,072	67.84
Total 2009-2013	494,702,276	59,962	82.50	242,127,846	2,599,190	93.16

	GFC	PRODES
Total Emitted C, 2009-2013, in Tg	494	242

Table S12
Estimated Emissions based on GFC and PRODES deforestation, 2002-2013, using the ABALB dataset.

Year	GFC Total C Emitted (tons)	Area Deforested (ha)	Average C emitted/ha	PRODES Total C Emitted (tons)	Area Deforested (ha)	Average C emitted/ha
2002	156,675,059	2,337,407	67.03	168,233,152	2,374,544	70.85
2003	142,736,436	2,040,730	69.94	216,281,482	2,813,478	76.87
2004	193,176,539	2,550,611	75.74	204,642,389	2,522,452	81.13
2005	198,199,007	2,484,337	79.78	176,787,340	2,191,494	80.67
2006	151,466,317	1,871,968	80.91	91,670,358	1,035,009	88.57
2007	120,116,576	1,506,616	79.73	99,717,954	1,092,151	91.30
2008	117,059,054	1,428,606	81.94	105,133,593	1,234,590	85.16
2009	90,868,234	995,513	91.28	55,451,919	588,034	94.30
2010	127,923,351	1,550,893	82.48	56,348,406	583,991	96.49
2011	88,786,174	1,081,183	82.12	52,798,213	524,333	100.70
2012	123,286,823	1,438,442	85.71	40,748,340	410,674	99.22
2013	80,345,764	958,081	83.86	50,691,706	507,666	99.85
Total	1,590,639,334	20,244,387	78.57	1,318,504,852	15,878,416	83.04
Total 2009-2013	511,210,346	6,024,112	84.86	256,038,584	2,614,698	97.92

	GFC	PRODES
Total Emitted C, 2009-2013, in Tg	511	256

Table S13

Differences in Differences Regression Results show association between use of PRODES as enforcement tool and decline in PRODES deforestation relative to GFC. The table contains results for a regression using raw deforestation data as the dependent variable instead of moving averages (A), with the moving averages but without longitude and latitude controls (B), with moving averages and latitude and longitude controls (C).

	A	B	C
T	-27.74***	-44.32***	-44.32***
	(6.034)	(5.416)	(5.051)
P	46.09***	99.63***	99.63***
	(10.20)	(9.250)	(8.626)
T X P	-110.6***	-92.41***	-92.41***
	(9.347)	(8.564)	(7.986)
Year	-35.64***	-48.75***	-48.75***
	(1.295)	(1.398)	(1.304)
Latitude			-0.000371***
			(0.00000396)
Longitude			0.000216***
			(0.00000257)
Constant	590.3***	668.4***	1788.3***
	(7.753)	(8.592)	(14.58)
Observations	118,344	98,620	98,620
R^2	0.028	0.036	0.162

Standard errors in parentheses

* $p < 0.05$, ** $p < 0.01$, *** $p < 0.001$

Tables S12

List of 12 GFC granules that cover the Legal Amazon of Brazil.

SN	GFC Granule ID
1	00N050W
2	00N060W
3	00N070W
4	00N080W
5	10N050W
6	10N060W
7	10N070W
8	10N080W
9	10S050W
10	10S060W
11	10S070W
12	10S080W

Table S13

Area and percent coverage of different forest and forest loss classes from analysis of GFC and PRODES forest cover and loss data in the Amazon Biome.

Land Type	Area (km^2)	%PRODES	%GFC
**Class 1**	111,822	73	51
**Class 2**	39,209	25	18
**Class 3**	2,738	2	1
**Class 4**	107,498	70	49
**Class 5**	345	0	0
**Total PRODES Forest loss (C1+C2+C3)**	153,768	100	70
**Total GFC Forest loss (C1+C4+C5)**	219,665	143	100
**PRODES Forest loss in both forests (C1+C2)**	151,031	98	69
**GFC Forest loss in both forests (C1+C4)**	219,320	143	100

Table S14

Area and percent coverage of different forest loss classes from analysis of GFC and PRODES forest cover and loss data in the legal Amazon.

Land Type	Area (km^2)	%PRODES	%GFC
**Class 1**	119,002	72	45
**Class 2**	43,494	26	16
**Class 3**	3,344	2	1
**Class 4**	145,908	88	55
**Class 5**	283	0	0
**Total PRODES (C1+C2+C3)**	165,840	100	63
**TOTAL GFC (C1+C4+C5)**	265,192	160	100
**PRODES FL in both forests (C1+C2)**	162,496	98	61
**GFC FL in both forests (C1+C4)**	264,910	160	100

Table S15

Spatial area of Class 4 in 30 m and a resampled 250 grid

Land Type	Area (km^2) on 30m grid	Area (km^2) when a majority filter (250m) was applied on the 30m grid	% Reduction
**Amazon Biome**	107,498	77,849	28
**Legal Amazon**	145,908	107,833	26
